# Factors affecting post-embolization fever and liver failure after trans-arterial chemo-embolization in a cohort without background infective hepatitis- a prospective analysis

**DOI:** 10.1186/s12876-015-0329-8

**Published:** 2015-08-04

**Authors:** Rohan Chaminda Siriwardana, Madunil Anuk Niriella, Anuradha Supun Dassanayake, Chandika Anuradha Habarakada Liyanage, Angappulige Upasena, Chandra Sirigampala, Hithanadura Janaka de Silva

**Affiliations:** 1 Faculty of Medicine, University of Kelaniya, Ragama, Sri Lanka; 2 Department of Radiology, North Colombo Teaching Hospital, Ragama, Sri Lanka

## Abstract

**Background:**

Transarterial-chemo-embolization (TACE) is used for palliation of unresectable hepatocellular carcinoma (HCC). We studied the tolerability of TACE in a cohort of patients with NASH and alcoholic cirrhosis related HCC.

**Methods:**

Of 290 patients with HCC (July 2011 - December 2014), 84 underwent TACE. They were monitored for post-TACE complications: postembolization fever (PEF), nausea and vomiting (NV), abdominal pain, infection, acute hepatic decompensation (AHD) and acute kidney injury (AKI).

**Results:**

84 patients [90.5 % males, 89.2 % cirrhotics, 89.2 % nodular HCC, median age 63 (34–84) years] underwent 111 TACE sessions. All were Child class A [69.4 % sessions (*n* = 77)] or B; ascites and portal vein invasion was present in 18 (16.2 %) and 15 (13.6 %), respectively.

42 (38.2 %) TACE procedures resulted in complications [PEF 28 (25.2 %), NV 4 (3.6 %), abdominal pain 9 (8.1 %), infection 7 (6.3 %), AHD 13 (11.7 %), AKI 3 (2.7 %)]. There were no immediate post-TACE deaths.

On univariate analysis elevated serum bilirubin (*p* = 0.046) and low serum albumin (*p* = 0.035) predicted PEF while low serum albumin (*p* = 0.021) and low platelet counts (*p* = 0.041) predicted AHD. In the multivariate model, factors with *p* < 0.200 on univariate analysis and factors derived from the previous literature were considered covariates. Female gender (*p* = 0.029, OR = 1.412), ascites (*p* = 0.030, OR = 1.212), elevated serum bilirubin (*p* = 0.007, OR = 4.357) and large tumour size (*p* = 0.036, OR = 3.603) were independent risk factors for PEF. Tumour diameter >5 cm (*p* = 0.049, OR = 2.410) and elevated serum bilirubin (*p* = 0.036, OR = 1.517) predicted AHD.

**Conclusion:**

In NASH and alcoholic cirrhosis related HCC patients pre-procedure serum bilirubin, ascites, tumour size and female gender predicted PEF post-TACE. Tumours larger 5 cm with elevated bilirubin predicted AHD post-TACE.

## Background

Hepatocellular carcinoma (HCC) is the fifth most common cancer worldwide and the third most common cause of cancer related deaths. Eighty to ninety percent of patients with HCC have underlying cirrhosis [1]. Globally, Hepatitis B (HBV) and Hepatitis C (HCV) are leading causes of cirrhosis and HCC. HBV and HCV is uncommon in Sri Lanka [2], and cases of cirrhosis are either alcohol related or non alcoholic steatohepatitis (NASH) related [2]. Although published data are not available, clinical impressions are that the incidence of cirrhosis and HCC are rising in the country.

Though there are many advances made in the treatment of HCC, the majority of tumours are diagnosed at an advance stage. As a result only a fraction of patients are eligible for curative therapy such as liver transplantation and resection [1]. Transarterial Chemo-embolization (TACE) and Radio Frequency Ablation are considered two of the most effective palliative treatments for inoperable HCC [3]. However, these patients need to be carefully selected to prevent complication for the best patient outcomes as a large majority of HCC patients have background cirrhosis. Hepatocyte injury during the procedure, ischaemic tumour necrosis and effects of chemotherapy can lead troublesome complication. Recognized complications of TACE include postembolization fever (PEF), nausea and vomiting (NV), right hypochondrial pain and infections. Occasionally, patients may develop life threatening complications such as acute hepatic decompensation (AHD) and acute kidney injury (AKI) [4–6].

This study investigated the tolerance and factors affecting tolerance to TACE in a cohort of patients with NASH and alcohol related cirrhosis with HCC.

## Methodology

A total of 290 consecutive individuals with alcoholic and NASH related cirrhosis complicated by the development of HCC, referred to the Hepato-Pancreatico-Biliary Unit of the Colombo North Teaching Hospital, Ragama, Sri Lanka, from July 2011 to December 2014 were included. 84/290 (29 %) patients underwent TACE; among these patients 111 TACE sessions were carried out.

Barcelona Clinic Liver Cancer (BCLC) tumour staging was followed to select patients suitable for TACE [7]. TACE was considered for unresectable intermediate-stage HCC (BCLC stage B or Child-Pugh class A/B with large or multifocal HCC, with no vascular invasion or extrahepatic tumour spread). However, selected patients with vascular invasion were also offered TACE after considering the extent of thrombosis and the patients’ general condition. Patients with complete portal vein thrombosis, high bleeding risk, total bilirubin level >3 mg/dl, Child-Pugh class C and extensive tumour involvement of more than 50 % of the liver were excluded.

Patients were given a single prophylactic dose of intravenous cefuroxime 750 mg and intravenous pantoprazole 40 mg one hour prior to the procedure. TACE procedure was performed by creating an arterial access at the groin under local anesthesia. The femoral artery was punctured using the Seldinger method. After selective canulation of the coeliac axis a 5 F/4 F Yashiro catheter was advanced into the relevant hepatic artery. The feeding artery to the tumour was selectively canulated using a micro catheter. Once the catheter was entered to the feeding artery doxorubicin 50 mg mixed with 10 ml Lipiodol was injected into the tumour. After this, 4-5 ml gelatin sponge was injected to occlude the feeding artery. The catheter was then withdrawn and a pressure dressing is applied.

During the immediate post procedure period, patients were monitored in the recovery area for any post procedure complications. Subsequently patients were transferred to the general ward. Regular antibiotics were only added to patients with clear evidence of infection. Anti-pyretics, analgesics and anti-emetics were not administered prophylactically and were only given if patients who developed PEF, pain or NV respectively. Patients with NV were treated with IV Ondensetrone 8 mg and PEF was treated with paracetamol. Duration and the frequency of these medications were decided depending on the response to treatment.

Patients were monitored for the development complications and the tolerability of the procedure clinically and biochemically. In post procedure day 1 and 3 a biochemical liver profile, prothrombin time, renal function tests and a full blood count were performed. Development of any post TACE complications such as PEF, NV, right hypochondrial pain, AHD and AKI were documented. In the absence of these complications, patients were discharged from hospital on post procedure day 3. Thereafter, patients were followed up at the out-patient clinic on post procedure day 7 with liver biochemistry, to detect delayed appearance of complications.

PEF was defined as a body temperature greater than 38.0 °C that developed within 3 days of TACE without evidence of infection [5]. ADH was define by the occurrence of clinical encephalopathy (West Haven grade 1 or higher), development of clinically detectable ascites or worsening of pre-existing ascites to clinically detectable or tense ascites, increasing prothrombin time by more than 3 s of the level prior to TACE, increase of serum bilirubin level to twice the level prior to TACE (4). AKI was defined as an abrupt increase in the serum creatinine level to over 50 % of the baseline level [8].

Factors that predict the two most common complications, postembolization fever (PEF) and acute hepatic decompensation (AHD), were separately assessed in a univariate analysis. Factors which had a p-value < 0.200 in the univariate analysis and important factors reported in previous literature were then taken into a multivariate logistic regression model. In the statistical analysis categorical variables were described using percentages and continuous variables were expressed using median and range. Comparison of categorical variables was made using chi square test and comparison of continuous variables was made using the *T* test and Mann–Whitney U- test where appropriate for an univariate analysis. Binary logistic regression was used for the multivariate analysis to find the most significant risk factors. A p < 0.05 was considered significant for all tests. Statistical analysis was performed using SPSS 20.

Ethical approval for the study was granted by the Ethics Review Committee of the Faculty of Medicine, University of Kelaniya, Ragama, Sri Lanka.

## Results and discussion

### Results

Eighty four patients underwent 111 TACE sessions during the study period. The median age of the population was 63 years (range 34–84). 90.5 % (*n* = 76) of the population were males. 89.2 % (*n* = 75) were cirrhotics. All patients were in either Child class A or B. 69.4 % (*n* = 77) of the TACE sessions were in Child class A patients. Majority (91.9 %) of the patients had a KF index above 80. Ascites was present in the CT scans of 16.2 % (*n* = 18) patients. Majority of the HCC were of the nodular type (*n* = 75; 89.2 %). Portal vein invasion was present in 15 (13.6 %).

Forty two (38.2 %) TACE sessions resulted in post-procedure complications. PEF was documented after 28 (25.2 %) sessions, NV after 4 (3.6 %), abdominal pain after 9 (8.1 %) and infection after 7 (6.3 %). AHD occurred following 13 (11.7 %) sessions and AKI after three (2.7 %). There were no fatalities related to the TACE procedure. There were no drop outs in this cohort. In absence of fatalities, all participants were reviewed day 7 post procedure: those with complications as in-patients and those discharged without complications as out-patients.

The univariate analysis indicated that a higher total bilirubin level (*p* = 0.046) and lower serum albumin level (*p* = 0.035) were possible risk factors for PEF while a lower serum albumin (*p* = 0.021) and platelet count (*p* = 0.041) were possible risk factors for AHD (Table 1). In the multivariate model, factors with *p* < 0.200 in the univariate analysis and important factors in the previous literature (4,8-11,14) were taken as covariates. Female gender (*p* = 0.029, 0R = 1.412), presence of ascites (*p* = 0.030, OR = 1.212), higher total bilirubin level (*p* = 0.007, OR = 4.357) and larger tumour diameter (*p* = 0.036, OR = 3.603) were independent risk factors for development of PEF following TACE. Tumour diameter >5 cm (*p* = 0.049, OR = 2.410) and higher bilirubin levels (*p* = 0.036, OR = 1.517) were independent risk factors for AHD (Table 2).

**Table 1:** Univariate analysis of factors effecting development of post embolization fever (PEF) and acute hepatic decompensation (AHD)

Variables	Patients with fever (*n* = 28)	Patients without fever (*n* = 83)	*p*-value	Patients with liver failure (*n* = 13)	Patients without acute liver failure (*n* = 98)	*p*-value
Age (years)	64 (37–84)	63 (34–78)	0.916	64 (37–84)	62 (34–76)	0.924
Gender (Males)	23 (84 %)	77 (93.2 %)	0.188	10 (83.3 %)	89 (91.2 %)	0.363
Diabetics	17 (55.9 %)	46 (55.6 %)	0.995	07 (55.6 %)	57 (58.3 %)	0.858
Body mass index (kg/m^2^)	25.03	23.88	0.124	24.18	24.33	0.842
KF index (<80 %)	03 (10 %)	03 (3.8 %)	0.198	02 (15.3 %)	08 (8.1 %)	0.199
Child Class B	09 (33.3 %)	14 (16.7 %)	0.252	04 (35.4 %)	28 (29.4 %)	0.743
Presence of ascites in computed tomogram	05 (17.9 %)	07 (8.1 %)	0.149	02 (15.3 %)	08 (8.1 %)	0.493
INR	1.23	1.15	0.546	1.21	1.16	0.451
Platelet count (/ml)	156×10^3^	171×10^3^	0.241	128×10^3^	177×10^3^	0.021
AST (U/L)	67.38	58.2	0.274	66.8	59.7	0.512
ALT(U/L)	46.1	48.6	0.703	46	48.3	0.782
Total Bilirubin (mg/dl)	1.39	0.71	0.046	1.35	0.87	0.094
Albumin (mg/dl)	3.30	3.51	0.035	3.14	3.49	0.041
Serum Creatinine (mg/dl)	1.11	0.89	0.321	1.07	0.92	0.235
Total tumour diameter (cm)	7.93	6.14	0.102	8.17	6.48	0.123
Type of HCC (diffuse %)	03 (1079 %)	03 (4 %)	0.311	02 (16.6 %)	72 (7.4 %)	0.292
Presence of Portal vein invasion	04 (16.2 %)	09 (11.3 %)	0.167	02 (16.6 %)	11 (11.9 %)	0.786

KF index-Karnofsky performance index, AST- aspartate amino transferase, ALT- Alanine amino transferase, INR- international normalized ratio

**Table 2:** Multivariate analysis of potential risk factors for development of post embolization fever (PEF) and acute hepatic decompensation (AHD)

Variables	Post-embolization fever (PEF) (*p*-value)	Odds ratio (95 % confidence interval)	Acute hepatic decompensation (AHD) (*p*-value)	Odds ratio (95 % confidence interval)
Gender^b^	0.029	1.142	-	
		(1.328-3.342)		
KF index (<80 %)	0.970	0.423	0.808	1.018
Child class^a^	-		0.571	0.449
Presence of ascites	0.030	1.212	0.850	0.712
		(1.023-2.231)		
Platelet count^a^	**-**		0.324	0.325
AST^b^	0.542	0.243	-	
Total bilirubin	0.007	4.357	0.036	1.517
		(2.443-6.219)		(1.112-4.536)
Albumin	0.126	0.564	0.060	0.965
Tumour diameter (>5 cm)	0.036	3.603	0.049	2.410
		(1.318-5.382)		(1.392-5.315)
Presence of portal vein invasion^a^	-		0.523	0.376
Type of tumour	0.437	0.785	0.134	0.879

KF index-Karnofsky performance index, AST- aspartate amino transferase

^a^- factors not included in multivariate analysis for PEF

^b^- factors not included in multivariate analysis for AHD

### Discussion

Our cohort of patients is unique in that none of them had cirrhosis secondary to infective hepatitis. In these patients with NASH and alcoholic cirrhosis related HCC, 38 % of TACE procedures resulted in complications; PEF (25.2 %) and AHD (11.7 %) were the commonest. There were no immediate post-TACE deaths. Female gender, tumour size over 5 cm, presence of ascites prior to the procedure and a high bilirubin level were predictors of post-TACE PEF. Lager tumour diameter and a high bilirubin level were predictors of AHD. The incidence of overall complications previously reported following TACE is about 30-70 % [6]. The commonest complication reported is PEF, occurring in 20-70 % in different settings [5, 9]. AHD has been previously reported in 5-20 % [4, 10, 11], infective complications, which include liver abscess and positive blood culture, in around 5 % [12], and upper gastrointestinal bleeding in 5-10 % of the patients following TACE [6].

The mechanism of PEF been extensively discussed. Most available evidence suggests that it is a result of collateral damage to normal liver tissue rather than tumour necrosis itself [13, 14], and hence does not influence survival. In a multivariate analysis, lipiodol dose and the post embolization AST level were predictors of PEF [9]. In another study, tumour size, AST level and lipiodol dose predicted PEF [6]. In our patients a standard dose of lipiodol was used in all cases. In our cohort we selected patients with a serum bilirubin less than 3 mg/dl. Even among them, a relatively higher bilirubin level and the presence of even mild ascites on abdominal CT independently predicted PEF. We noted an association between PEF and tumours larger than 5 cm. Data from previous studies does not support this finding [5]. We cannot explain why being female seemed to predict PEF. Though PEF is self-limiting and has no impact on post procedure survival, the complication is important for pre-procedure patient counseling.

AHD and liver failure are dreadful complications that lead to significant morbidity and mortality. Higher bilirubin levels indicate poor background liver function and we found that a large tumour diameter and a high bilirubin level predicted of AHD. Similar findings have been reported by others [4, 10], and TACE in patients with tumours larger than 5 cm has been associated with post procedure liver failure. In our cohort there were no deaths due to AHD and liver failure and all patients were successfully managed medically. However, hospital stay was prolonged up to one to two week. Though a higher serum bilirubin and tumour size should not be contraindications, such patients should be warned, and prior anticipation and precautions may reduce AHD and its progression to liver failure.

Minority of patients developed complications such as NV, abdominal pain, infection and AKI. These were transient and responded well to standard treatment. NV was managed with anti-emetics. Abdominal pain responded well to simple analgesics such as paracetamol, not exceeding total daily dose of 2 g per day. This dose was well tolerated by the patients. Infections were treated with intravenous ceftriaxone 1 g twice daily for three 3 to five days. There were no cases of confirmed spontaneous bacterial peritonitis. AKI was transient and responded well to rehydration. None required treatment escalation to include volumes expansion, vasoconstrictors or renal replacement therapy.

We did not encounter upper gastro intestinal bleeding post-TACE. In previous literature this is reported to occur in about 5-10 % [6]. As a routine practice we eradicated varices in all our patients before undertaking TACE and all patients were on low dose proton pump inhibitors before and after the procedure, which may have played a role in this this complication not developing in our patients.

Relative short term follow up period of this study is a limitation. Therefore, observation for the development of delayed complications such as hepatic abscess was not possible in this study.

Careful selection of patients for TACE in the setting of HCC, will likely reduce anticipated complications of this procedure. The predicator of such complications, from the present study and as well as from previously published studies, should guide clinicians for early detection and treatment of post-TACE complications.

## Conclusion

In this cohort of HCC patients with NASH and alcohol related cirrhosis, 38 % of TACE procedures resulted in complications, of which PEF and AHD were the commonest. Female gender, pre-procedure serum bilirubin, ascites, and tumour size predicted PEF post-TACE. Tumours larger than 5 cm and elevated serum bilirubin predicted the development of AHD post-TACE. In this cohort there were no fatalities related to TACE.

## Abbreviations

(HCC): Hepatocellular carcinoma
(TACE): Transarterial-chemo-embolization
(PEF): Postembolization fever
(NV): Nausea and vomiting
(AHD): Acute hepatic decompensation
(AKI): Acute kidney injury
(HBV): Hepatitis B
(HCV): Hepatitis C
(NASH): Non alcoholic steatohepatitis
(BCLC): Barcelona Clinic Liver Cancer
